# Health-related quality of life among adults with diverse rare disorders

**DOI:** 10.1186/s13023-017-0730-1

**Published:** 2017-12-07

**Authors:** Kathleen R. Bogart, Veronica L. Irvin

**Affiliations:** 1 School of Psychological Science, Oregon State University, 2950 SW Jefferson Way, Corvallis, OR 97331 USA; 2 College of Public Health and Human Services, Oregon State University, 2250 SW Jefferson Way, Corvallis, OR 97331 USA

**Keywords:** Quality of life, Rare disease, Rare disorder, PROMIS, Anxiety, Depression

## Abstract

**Background:**

Twenty-five to 30 million Americans live with a rare disease (RD) and share challenges unique to RD. The majority of research on RDs has focused on etiology, treatment and care, while the limited health-related quality of life (HRQL) research has been restricted to single RDs, small samples, or non-validated measures. This study reports HRQL among adults with diverse RDs, and compares their scores to those of the U.S. population and people with common chronic health conditions.

**Methods:**

We conducted a cross-sectional survey of adults living in the U.S. diagnosed with any RD. Participants were recruited through RD organizations and completed the online survey between December 2016 and May 2017 (*n* = 1218). HRQL was assessed using the standardized Patient-Reported Outcomes Measurement Information System (PROMIS). RDs were classified into categories defined by Orphanet. Means and 95% confidence intervals were calculated for the main sample and for RD categories and were compared to published U.S. population norms and common chronic disease norms. Intercorrelations were conducted between HRQL, demographics, and RD experiences.

**Results:**

When compared to the norms for the U.S. population and for those with common chronic diseases, mean HRQL scores were significantly poorer across all six PROMIS domains for the main sample, and were usually poorer when analyzed by sub-sets of specific RD classifications. People with rare systemic and rheumatologic, neurological, and immune diseases had the poorest HRQL. Participants had poorer HRQL if they had multiple RDs, lower income, were female, or older. Having symptoms longer was associated with worse HRQL, however, having a formal diagnosis longer was associated with better HRQL.

**Conclusions:**

This study is the first to examine HRQL in a large, heterogeneous sample of RDs using validated measures. There is a significant disparity in HRQL among people with RD compared to the general population and people with common chronic diseases. Poor HRQL could be attributed to challenges accessing diagnoses, medical information, treatment, psychosocial support, and coping with stigma and uncertainty. As most individuals with RDs will not be cured in their lifetimes, identifying ways to improve HRQL is crucial to patient-centered care and should be a funding priority.

## Background

Rare diseases and disorders (RD) are defined in the U.S. as affecting fewer than 200,000 people per year; thus approximately 25–30 million Americans have a RD [1]. Although there are about 7000 different RD [1], people with diverse RDs share similar challenges [2–4]. Despite differences in disease etiology and symptoms, many RDs are chronic, involve multi-system dysfunction, have no effective treatment, and require complex care [4, 5]. It is well established that chronic diseases present challenges to health-related quality of life (HRQL) [6], an individual’s perceived physical and mental well-being [7]. However, RDs may create additional threats to HRQL due to poor access to information, treatment, and support [2, 3, 8], combined with high levels of stigma [9, 10].

The experience of living with a RD often begins with a struggle to find a diagnosis. Persons in the U.S. experience an average diagnostic delay of 7 years [11]. Once diagnosed, people quest for doctors with expertise and information they can trust [3, 4, 8, 11]. For this reason, they turn to RD organizations as their primary source of information and support [4]. The chronic pain, fatigue, and physical impairments associated with many RDs are exacerbated by challenges in finding support and treatment, and the fact that most RDs have no effective treatment or cure [5]. The rarity of the condition may mean that informal and formal members of one’s support system do not know how to provide appropriate support, or they may even question the legitimacy or severity of the condition because it is not well understood [12]. Indeed, two thirds of adults with RD do not receive sufficient medical, informational and psychosocial support [3, 11]. Similarly, a lack of information about one’s disease, its management, and uncertainty about prognosis and course may result in anxiety and depression [11]. RDs are stigmatizing because they are isolating (i.e. many people with RD never have the normalizing opportunity to meet others like themselves) [3] and other people lack awareness, meaning people with RD are frequently misunderstood, avoided, and blamed [9, 13]. Social and environmental barriers like stigma limit ability to participate in social roles and activities [13].

A few previous studies of RD HRQL have focused on specific RDs, including systemic sclerosis/scleroderma (a rare skin disease) [14], neurofibromatosis (a rare developmental anomaly) [15], osteogenesis imperfecta (a rare bone disease) [16], and Huntington’s disease (a rare neurological disease) [17], and found consistently poor HRQL according to the Patient-Reported Outcomes Measurement Information System (PROMIS), a measurement tool developed with the U.S. National Institutes of Health (NIH) to measure HRQL [18]. For example, people with pulmonary fibrosis, a rare respiratory disease, were found to have high levels of depression (commensurate with individuals diagnosed with major depression), and anxiety scores higher than individuals with chronic obstructive pulmonary disease, a common respiratory disease [19].

The few RD HRQL studies have been restricted by focusing on a single, specific disease, resulting in small samples. Given the similarities in experience across RDs described above, examining heterogeneous RDs would add crucial new information about commonalities and differences in HRQL across RD types and will allow for sufficient statistical power. Indeed, the major RD organizations, which exist as umbrella organizations to support people with all RDs, including National Organization for Rare Disorders (NORD), Orphanet, and EURORDIS, already consider RDs collectively when providing advocacy, support, education, and funding for RDs. It is also useful to examine HRQL across broad categories of RDs (e.g. neurologic, neoplastic, etc.), because research funding and policy is often distributed according to these categories (i.e. at NIH). Understanding HRQL in RDs and RD categories can inform the support and funding priorities provided by these organizations. As such, researchers have examined RDs collectively to understand experiences in healthcare and HRQL [8, 11]. There only previous examination of collective RD HRQL, a report published by Shire, which found that 86% of Americans with a variety of RDs stated that they thought their RD caused anxiety symptoms and 75% stated it caused depression symptoms [11]. Their findings suggest that people with diverse RDs face significant risk of HRQL problems. However, this report surveyed only 144 adults in the U.S. with RD and did not use validated measures [11]. The purpose of the present study was to quantitatively describe HRQL among adults living in the U.S. with a variety of RDs and RD categories using PROMIS, and to compare their scores to those of the general population and people with common chronic health conditions. We predicted that participants with RD would show worse HRQL compared to population norms and chronic health condition norms.

## Method

### Study design and participants

The current project is a cross-sectional survey focused on adults (18 or older) in the U.S. with any RD. No complete sample frame of adults with RD in the U.S. exists. For this reason, RD organizations, the primary source of information and support for individuals with RD [4], were enlisted to recruit participants for this study. NORD, the major U.S. umbrella organization for individuals with RD and the organizations that serve them, shared recruitment information with all 242 of its member organizations, which in turn, distributed information to their networks via newsletters, email, and social media. Coordination of Rare Diseases at Sanford shared recruitment information with all adult members of its registry (2006 individuals). Recruitment information was also shared by other individuals and organizations through snowball sampling.

### Procedure

The survey was conducted primarily online to maximize accessibility to this geographically dispersed population. Participants followed a link to the survey administration website Qualtrics, an encrypted, password-protected platform, between December 2016 and May 2017. Mailed paper surveys were available by request (*n* = 14 participants submitted a paper survey). If it was difficult for a participant to enter responses, they were permitted to dictate their responses to another person to enter for them (*n* = 24 did so). Approximate time to complete the survey was 40 min.

### Measures

The PROMIS Profile is comprised of independent domains measuring quality of life in physical, mental, and social health [20]. We selected six domains (i.e. anxiety, depression, fatigue, pain interference, physical function, and ability to participate in social roles and activities). As discussed in the introduction, these domains are frequently described in the RD HRQL literature, and we anticipated that they would be common challenges in a broad sample of RDs. Four-item short forms were used for all domains. Following PROMIS scoring guidelines, domains were scored using the published T-scores calibrated such that a *M* of 50 and an *SD* of 10 is representative of the U.S. general population [18]. Higher numbers indicate greater amounts of the domain. As part of the PROMIS calibration sample, norms were also published for participants with 24 common chronic diseases [6]. This allows for score comparisons to the general population as well as people with common chronic diseases. Norms for common chronic diseases on the ability to participate in social roles and activities scale are not available because this scale was created after the development of those norms.

We also examined the following demographic information and information about experiences with RD: age, gender, race, income, country of residence, diagnosed RD name, number of RDs, RD name, duration of symptoms, and years since diagnosis.

This data was collected as part of our larger Adults with Rare Disorder Support (AWaRDS) Study, and additional measures were collected for the purposes of other research not described here. Survey items are available upon request.

### Analysis plan

We conducted analyses using IBM SPSS Statistics 24 first on the main sample of RDs and then replicated the analyses with a subset of participants based on their RD categories. Variables were examined for normality using Q-Q plots.

#### Main sample of RDs

In order to understand the interrelationships between demographic factors and PROMIS scores, Pearson correlation coefficients were calculated. 95% confidence intervals for each PROMIS scale mean for the main sample of RDs were calculated in order to determine whether they differed from population norms and common chronic disease sample means. The criterion for statistical significance was CI’s that did not include the population or common chronic disease means (equivalent to a one-sample t test). Although there has been little research on what constitutes minimal clinically important differences for most PROMIS subscales, 1 *SD* has been suggested as a rule of thumb [15]. More specific minimal clinically important difference estimations are available for anxiety, depression, and fatigue (0.3–0.5 *SD*), pain (0.4–0.6 *SD*), and physical function (0.2 *SD*) [21, 22].

#### RD categories

We also compared scores for RD categories to the population mean and common chronic disease samples. The International Classification of Disease system is not recommended for RDs [23]; instead, Orphanet’s linearization rules [24] and their database of categories [25] were used to assign each RD to a single category. A power analysis indicated that a sample of 34 was needed to achieve 80% power with a medium effect size (*d* = .5), so any category that contained more than 34 participants was compared to the population and common chronic disease norms by inspecting the 95% CIs.

## Results

A total of 1473 participants completed the survey. Twelve were excluded because of duplicate entries (ascertained by duplicate email addresses). Researchers then confirmed self-reported RDs were rare according to the NIH definition using their Genetic and Rare Disorder Information Center database [26]. Eighty-one participants were excluded because they indicated they were undiagnosed, did not specify the name of a RD, or their disease was not classified as rare. Although the project was based in the U.S., 162 participants from outside of the U.S. responded. These were not included in the following analyses in order to allow comparisons to U.S. population norms. Thus, a total of 1218 participants were included in the main sample for this study. Participant characteristics are shown in Table 1. Information can also be gleaned from the combination of demographic information collected. The difference between the average length of time from symptom onset to diagnosis (as shown in Table 1) indicates that participants in this sample experienced a diagnostic delay of 9 years. Calculating the difference between participant age and length of time since diagnosis revealed that 106 participants (9%) were diagnosed with a RD before age 18.

**Table 1:** Sociodemographic and rare disease characteristics among 1218 participants in the U.S

Characteristic	Frequency	Percent	M (SD)	Range
Age			51.50 (14.56)	18–89
Duration of RD symptoms in years			18.84 (16.49)	0–75
Years since RD diagnosis			9.76 (11.46)	0–70
Number of RDs perparticipant				
1	1058	87%		
2	130	11%		
3	22	2%		
4	4	<1%		
5	4	<1%		
Gender				
Female	935	77%		
Male	279	23%		
Other	4	<1%		
Income				
under $10,000	58	5%		
$10,000–20,000	95	8%		
$20,001–30,000	118	10%		
$30,001–45,000	127	11%		
$45,001–60,000	161	14%		
$60,001–75,000	128	11%		
$75,001–90,000	107	9%		
$90,001 and above	376	32%		
Race/ethnicity				
American Indian orAlaska native	11	1%		
Asian	23	2%		
Black or African American	13	1%		
Hispanic or Latino/a	31	3%		
Native Hawaiian orPacific Islander	2	6%		
White	1149	94%		
Other	31	3%		

Percentages do not add to 100 due to rounding

A total of 232 RDs were represented. The 10 most commonly endorsed RDs were: ataxia (neurologic) *n* = 150, Waldenstrom macroglobulinemia (neoplastic) *n* = 86, mastocytosis (neoplastic) *n* = 84, Ehlers-danlos syndrome (systemic and rheumatological) *n* = 75, mast cell activation disorder (immune) *n* = 70, idiopathic hypersomnia (neurologic) *n* = 67, narcolepsy (neurologic) *n* = 63, cutaneous T-cell lymphoma (neoplastic) *n* = 44, Bell’s palsy (neurologic) *n* = 29, and erythromelalgia (neurologic) *n* = 29. Table 2 shows the number of RDs falling into each Orphanet classification.

**Table 2:** Rare disease classifications among 1218 participants with rare diseases in the U.S

Orphanet Classification	Frequency	Percent
Bone	19	1%
Cardiac	3	<1%
Developmental defect	71	5%
Endocrine	52	4%
Eye	3	<1%
Gastroenterologic	11	1%
Hematologic	27	2%
Hepatic	12	1%
Immune	93	7%
Inborn errors of metabolism	31	2%
Infectious	16	1%
Neoplastic	268	19%
Neurologic	581	41%
Otorhinolaryngologic	41	3%
Psychiatric	2	<1%
Renal	5	<1%
Respiratory	14	1%
Skin	52	4%
Systemic and rheumatological	118	8%
Urogenital	1	<1%
Total	1420	100

### Main RD sample analyses

Table 3 shows the intercorrelations between participant characteristics and outcome variables. For the purpose of these correlations, due to the small number of persons of color, they were grouped together to analyze race as coded as person of color = 0, white = 1. Gender was coded as female = 0 and male = 1. Older individuals had less fatigue, less pain, less anxiety, less depression, and less physical function. Females had greater fatigue, pain, anxiety, and depression, and less ability to participate in social roles and activities. Having symptoms of one’s RD longer was associated with greater fatigue, pain, less ability to participate, and less physical function. However, having one’s diagnosis longer was associated with lower fatigue, anxiety, and depression, and better ability to participate in social roles and activities. Having multiple RDs was associated with greater fatigue and pain, less ability to participate, and less physical function. Higher income was associated with lower fatigue, pain, anxiety and depression, and better ability to participate and physical function.

**Table 3:** Intercorrelations of socio-demographic and rare disease characteristics and Health-Related Quality of Life outcomes in 1218 participants

	Age	Gender	Duration of symptoms	Years since diagnosis	Number of rare diseases	Race	Income	Fatigue	Pain interference	Anxiety	Depression	Physical function
Gender	.23^**^											
Duration of symptoms	.07^*^	−.07^*^										
Years since diagnosis	.12^**^	0.03	.58^**^									
Number of rare diseases	−.08^**^	−0.05	.10^**^	.06^*^								
Race	.03	−.09^**^	.01	.03	.03							
Income	.19^**^	.09^**^	−.13^**^	.05	−.06	.09^**^						
Fatigue	−.29^**^	−.21^**^	.08^**^	−.12^**^	.17^**^	.01	−.20^**^					
Pain interference	−.08^**^	−.12^**^	.15^**^	.00	.19^**^	−0.03	−.22^**^	.53^**^				
Anxiety	−.30^**^	−.19^**^	−.01	−.10^**^	.03	−.04	−.20^**^	.47^**^	.38^**^			
Depression	−.25^**^	−.15^**^	.01	−.08^**^	.03	−.03	−.20^**^	.53^**^	.40^**^	.76^**^		
Physical function	−.07^*^	.00	−.07^*^	.01	−.12^**^	.03	.21^**^	−.45^**^	−.51^**^	−.25^**^	−.34^**^	
Ability to participate in social roles	.05	.11^**^	−.08^**^	.08^**^	−.17^**^	−0.03	.17^**^	−.65^**^	−.54^**^	−.39^**^	−.48^**^	.72^**^

Race was coded as person of color = 0, white = 1. Gender was coded as female = 0 and male =1

* *p* < .05, ** *p* < .01

**Fig. 1:**
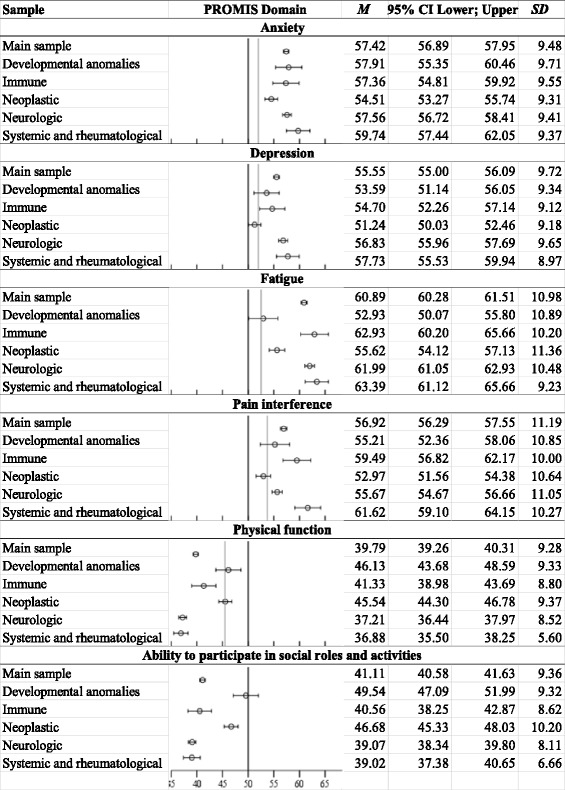
PROMIS mean scores for main sample of persons with RD and by RD category. Error bars represent 95% CI. Black lines indicate U.S. population mean, and gray lines indicate means for common chronic diseases. [6, 18] Higher numbers mean higher amounts of the measured construct, so high scores on anxiety, depression, fatigue, and pain interference indicate poor HRQL, while low scores on physical function and ability to participate in social roles and activities means poor HRQL. Main sample *n* = 1218; neurologic *n* = 480; neoplastic *n* = 221; systemic and rheumatological *n* = 66; developmental anomalies *n* = 58; and immune *n* = 56

Figure 1 shows the PROMIS *M*, *SD*s, and 95% CIs for the main sample. Participants in the main RD sample had poorer average HRQL on all PROMIS scales compared to population norms and common chronic disease norms.

### RD category analyses

Next, in order to analyze RD categories, it was necessary to exclude 160 participants who selected more than one RD from this analysis because they may fall into multiple categories. The following five classifications met the power requirements to be analyzed as separate RD categories: neurologic (*n* = 480), neoplastic (*n* = 221), systemic and rheumatological (*n* = 66), developmental anomalies (*n* = 58), and immune (*n* = 56).

Figure 1 also shows that most RD categories scored significantly worse than the population norm. The only exception was that persons with developmental anomalies did not differ from the U.S. norm on ability to participate in social roles and activities. When comparing RD categories to common chronic disease norms, most RD classifications scored significantly worse. A few RD categories did not differ from common chronic disease norms on the following scales: 1) pain scores among persons with neoplastic and developmental anomalies; 2) fatigue scores among persons with developmental anomalies; 3) depression scores for persons with developmental anomalies and neoplastic diseases; and 4) and physical function among persons with developmental anomalies and neoplastic diseases.

## Discussion

In the largest study of diverse RDs, we examined the HRQL of individuals with RD in the U.S. compared to the general population and individuals with common chronic illnesses. Findings were remarkably consistent: compared to both sets of norms, our main sample of persons with RD as a whole scored consistently poorer on every subscale, and those scores were clinically important differences for all but one scale (ability to participate in social roles and activities). Although it is well documented that individuals with common chronic diseases experience challenges to HRQL [6], our findings show that, as predicted, the experience of living with RD leads to even greater HRQL threat. RDs come with a number of unique challenges including accessing diagnoses, medical information, treatment, psychosocial support, and coping with stigma and uncertainty [3, 9, 11].

Intercorrelations revealed interesting patterns. Women experienced poorer HRQL than men. Older participants showed a somewhat paradoxical pattern of poorer physical function yet lower anxiety, depression, fatigue, and pain, which has been found in other chronic disorders [27, 28]. This may be attributed to the notion that physical function limitations are more expected in older age and may result in less distress than when the occur in younger individuals [28]. Interestingly, having symptoms of one’s disease longer was associated with poorer HRQL, but having had one’s diagnosis for longer was associated with better HRQL. Participants in our sample experienced long diagnostic delays, averaging 9 years. This pattern of findings suggests that, although experiencing symptoms for an extended period of time is a risk factor for poor HRQL, receiving a diagnosis is a gateway to treatment and support that can, over time, alleviate some of the HRQL challenges of having a RD. People navigating more than one RD experienced poorer HRQL, suggesting that RDs have an additive effect. People with higher income experienced better HRQL in all domains. People with higher HRQL are more likely to be employed, generate income, have private insurance, and access quality healthcare. Higher income also affords the ability to travel greater distances to seek expert care and support.

Examining specific RD categories, participants with systemic and rheumatic diseases had the poorest HRQL profile, with the worst scores on every domain and scores at least one *SD* worse than the norm on every scale. Neurological diseases were also characterized by very poor HQRL, with poor physical function, fatigue, and ability to participate in social roles and activities (all greater than 1 *SD* from the norm). Poor HRQL was also found in immune diseases, including fatigue and pain scores (1 *SD* from the norm). Participants with neoplastic diseases did not show an extreme pattern of HRQL deficits, with no scores 1 *SD* or greater from the norm.

Participants with developmental anomalies experienced fewer HRQL deficits than the other categories. In fact, they did not differ from the general U.S. population in ability to participate in social roles and activities, and they did not differ from common chronic diseases in most domains. These findings may be in line with previous research which has found that people with congenital or early onset disabilities and RDs are better adapted and have better disability self-efficacy than those with acquired conditions [29]. Persons with congenital or early onset RDs have had a long time to adapt, and went through their cognitive, physical, and social development with their RD conditions [29]. Thus, they may not experience a functional loss and are less likely to have experienced a change in identity [29].

Our study is noteworthy for its large, diverse sample of people with RDs and use of PROMIS to make comparisons to representative population norms. However, our findings should be considered in light of certain methodological limitations. As with most RD research, it is not possible to determine the representativeness of our sample because there is little research on the demographics of people living with RD [8]. Further, the U.S. does not track diagnoses of most RDs [1], so it is not possible to assess whether certain RDs were over- or under- represented in our sample. For this reason, RD prevalence estimates may change and categorization of diseases as rare or not may change over time. Additionally, our sample had higher income than the general U.S. population. As higher income is associated with higher quality of life in our sample and others [30], and the population of people with RD may be less connected to RD networks and have lower income than our sample, it stands to reason that the population of people with RD as a whole may have *even poorer* HRQL than our results indicate. It should be noted that the second largest study of individuals with diverse RDs (*n* = 810), which was conducted in Australia using similar sampling and data collection methods, had a number of similar demographics: a high number of people living with multiple RDs (16%), a high number of neurological RDs, about three quarters of their sample was female, and the majority were middle-aged.

Another consideration is the heterogeneity in RD experiences that may result from our efforts to sample diverse RDs. For example, a participant with cutaneous T cell lymphoma who is relapsing would likely have poorer HRQL than someone who is in remission. That participants scored, on average, significantly poorer in HRQL compared to non-RD samples despite this heterogeneity, strengthens our conclusion that people with RDs as a whole are at greater risk of HRQL problems. This suggests that even people with mild or remitting symptoms need more support than those with common diseases. The final limitation is that Orphanet acknowledges that their linearization rules for categorizing diseases are sometimes somewhat arbitrary [24]. For example, according to their rules, endocrine tumors are classified as rare neoplasms rather than endocrine disease, even though the RD has features of both categories [24]. For this reason, nuances between RDs and categories may have been missed.

## Conclusions

There is a paucity of research on and interventions for HRQL in people with RD. People with RDs strongly desire to meet others with their condition [31]. but most have never done so [3]. Research on the RD Moebius syndrome suggests that offering ways for people with RDs to gather for social support may buttress quality of life by reducing stigma, increasing knowledgeability about the RD, and reducing isolation [32, 33]. Our group is currently examining the support needs of people with RD to better understand unique challenges and identify sources of resilience to be built upon.

RD HRQL disparities are driven by insufficient funding and infrastructure for research, treatment, and psychosocial support; there is nothing inherent in the pathology of RDs that creates a greater challenge to HRQL than a common chronic disease. A number of RD organizations target important challenges such as identification of new treatments and cures, understanding causal pathways, providing information to patients, caregivers, and providers, and lobbying for policy conducive to orphan drug discovery. However, few organizations prioritize HRQL issues like psychosocial support. For example, none of the Request for Applications (RFAs) funded by the NIH’s Rare Disease Clinical Research Network included any objectives or priorities to assess psycho-social support or outcomes [34, 35]. All RFAs were focused on genetic and epidemiological studies or clinical trials for diagnostic or treatment services. As most individuals with RDs will not be cured in their lifetimes [5], identifying ways to improve HRQL is of utmost importance to patient-centered care. For these reasons, we recommend that RD HRQL should be included in mission statements and funding priorities of health agencies and RD organizations. This may be of particular importance for rare systemic and rheumatologic, neurological, and immune diseases, which involved the most significant HRQL challenges. These efforts should especially target women and individuals with low income, who experienced the most HRQL deficits.

## Abbreviations

AWaRDS: Adults with Rare Disorders Support study
HRQL: Health-Related Quality of Life
NIH: National Institutes of Health
NORD: National Organization for Rare Disorders
PROMIS: Patient-Reported Outcomes Measurement System
RD: Rare disease or disorder
